# Supplementary material for: Serum Biomarkers in Connective Tissue Disease-Associated Pulmonary Arterial Hypertension
Source: Int J Mol Sci. 2023 Feb 20;24(4):4178. doi: 10.3390/ijms24044178 (PMC9967966; doi:10.3390/ijms24044178)
Supplement: Supplementary file 1 [file ijms-24-04178-s001.zip › ijms-2165250-supplementary.pdf]

Supplementary Table S1. Comparison between serum biomarkers in CTD-PAH vs. IPAH.

Biomarker	CTD-PAH	IPAH
ET-1	Increased [32, 34, 35, 36, 37, 39]	Increased [32-33]
GDF-15	Increased [53]	Normal range [53]
Eng	Increased [56]	Not investigated
FSTL3 and MDK	Increased [59]	Not investigated
Chemerin	Increased [60]	Not investigated
RAGE, MMP-2, NP-1	Increased [61]	Not investigated
Pro-MMP-10	Increased [62]	Not investigated
TIMP-4	Increased [63]	Increased as in CTD-PAH [64]
OPN	Increased [70]	Increased [178]
UA	Increased [23, 75, 76]	Increased [72, 73]
VEGF165A	Increased [79]	Increased [79]
PlGF	Increased [79]	Normal range [79]
ES	Increased [79]	Lower levels than CTD-PAH [79]
vWF	Increased [85, 86]	Increased [84]
TM	Increased [90]	Decreased [91]
Autoantibodies		n/a
IL-1β, IL-6	Increased [85, 140,141]	Increased [139]
IL-18BP	Increased [144]	Not investigated
IL-32	Increased [145]	Normal range [145]
PTX3	Increased [146]	Increased [146]
Chemokines (CXCL4, CXCL16, CCL20, CCL21]	Increased [150-154]	Not investigated
BNP- NTproBNP	Increased [23, 162, 163]	Increased (but lower levels compared to SSc-PAH) [164]
Cardiac troponin	Increased [173,174]	Increased [172]

BNP, brain natriuretic peptide; CCL, C-C motif ligand; CXCL, C-X-C motif ligand; CTD, connective tissue disease; Eng, endoglin; ES, endostatin; ET-1, endothelin-1; FSTL-3, follistatin-like 3; GDF-15, growth differentiation factor-15; IL, interleukin; IPAH, idiopathic pulmonary arterial hypertension; MDK, midkine; PAH, pulmonary arterial hypertension; MMP, matrix metalloproteinase; n/a, not applicable; NP-1, neuropilin-1; NT, N-terminal; OPN, osteopontin; PlGF, placental growth factor; PTX3, pentraxin 3; RAGE, receptor for advanced glycation end products; TIMP, tissue inhibitor of metalloproteinase; TM, thrombomodulin; UA, uric acid; VEGF, vascular endothelial growth factor; vWF, von Willebrand factor.
